# Alcohol and Stress in the Military

**DOI:** 10.35946/arcr.v34.4.04

**Published:** 2012

**Authors:** Jeremiah A. Schumm, Kathleen M. Chard

**Affiliations:** **Jeremiah A. Schumm, Ph.D.,***is a clinical psychologist at the Posttraumatic Stress Disorder and Anxiety Disorders Division, Cincinnati Veterans Affairs Medical Center and an assistant professor of Clinical Psychiatry at the University of Cincinnati, Cincinnati, Ohio.*; **Kathleen M. Chard, Ph.D.,***is director, Posttraumatic Stress Disorder and Anxiety Disorders Division, Cincinnati Veterans Affairs Medical Center, and associate professor of Clinical Psychiatry, University of Cincinnati, Cincinnati, Ohio.*

**Keywords:** Alcohol consumption, alcohol use and abuse, problematic alcohol use, stress, stress reaction, posttraumatic stress disorder, military personnel, active military, veteran, combat, military sexual trauma, causal pathways, self-medication, genetic vulnerability, co-occurring disorders

## Abstract

Although research has independently linked stress experienced by military personnel to both alcohol use and posttraumatic stress disorder, more recently researchers have noted that there also is a significant overlap between stress reactions and alcohol use in veterans and active-duty service members. This overlap seems to be most understood in individuals who have experienced combat or military sexual trauma. This article will provide a brief review of some potential causal mechanisms underlying this relationship, including self-medication and genetic vulnerability models. It also addresses the possible implications for assessment and treatment of military personnel with co-occurring disorders.

Problematic alcohol use within the United States military has been linked to substantial financial and productivity losses. Data from 2006 revealed that excessive alcohol consumption cost the U.S. military $1.12 billion per year (Harwood et al. 2009). Regarding medical expenditures, studies have found that excessive alcohol use by military members results in an annual cost of $425 million. Excessive drinking within the military is estimated to result in a loss of 320,000 work days and 34,400 arrests per year, half of which are for driving under the influence. Finally, these data indicate that each year excessive alcohol use results in 10,400 active-duty military being unable to deploy and 2,200 being separated from service duty. Given the substantial cost of alcohol misuse, it is imperative to examine factors that may contribute to problematic drinking so that interventions can be employed to address this issue within the military.

This article will examine the links between military traumatic stress and mental health problems, such as post-traumatic stress disorder (PTSD) and between military traumatic stress and problematic alcohol use. Furthermore, it will summarize the pathways that may explain these links and describe possible implications for assessment and interventions with veterans.

## Prevalence of Problematic Alcohol Use in the U.S. Military

Frequent heavy drinking, defined as consuming five or more drinks on one or more occasions per week, occurs among a substantial proportion of U.S. military personnel and varies as a function of military demographic characteristics. In a large-scale survey, Bray and Hourani (2005) found that the prevalence of frequent heavy drinking in the military from 1980 through 2005 ranged from 15 to 20 percent. Consistent with findings from civilian samples that show gender differences in rates of heavy drinking, military men were nearly 3.5 times more likely to report frequent heavy drinking compared with women in the military. Frequent heavy drinking also varied as a function of ethnicity, with Hispanic and non-Hispanic Whites exhibiting higher rates of problematic drinking than non-Hispanic Blacks. In addition, military rank significantly correlated with frequent heavy drinking; rates were six times greater among enlisted personnel with the lowest rankings compared with officers. Rates of heavy drinking also varied as a function of military service branch, with those in the Army, Navy, and Marines being more likely to report frequent heavy drinking than those in the Air Force. Other population-based studies of the U.S. military have found that heavy drinking is more likely to occur among younger military members (Stahre et al. 2009). Together, these results suggest that certain military demographic groups (e.g., younger, low-ranking, non–Air Force, White or Hispanic men) may be especially prone to engage in frequent heavy drinking.

Young adults in the military are more likely than their civilian counterparts to engage in heavy drinking. For example, Ames and Cunradi (2004) found that rates of heavy drinking were significantly higher among male military personnel aged 18 to 25 years (32.2 percent) compared with male civilians in a similar age range (17.8 percent). The researchers also found significantly elevated rates of heavy drinking among women in the military compared with similarly aged female civilians (5.5 percent). In addition to demographic factors, military-related stressful events also may contribute to the high rates of problem drinking observed.

Alcohol misuse also frequently occurs among a substantial proportion of combat veterans. In one population-based study of 88,235 veterans returning from Operation Iraqi Freedom (OIF), Milliken and colleagues (2007) found that 12 to 15 percent of veterans endorsed problematic alcohol use in the 3 to 6 months following their return from combat. These data suggest that alcohol misuse occurs among a substantial number of veterans who are exposed to combat-related traumatic stress and highlight the importance of understanding the relationships between stressful military experiences (e.g., combat and military sexual trauma) and alcohol misuse.

## Military Trauma and Stress-Related Disorders

Stress-related disorders in response to military service have been noted throughout history. Whether labeled “combat fatigue” or “shell shock” or PTSD, there have been consistent reports in the literature documenting that exposure to combat experiences can lead to an impairment of psychological functioning in military personnel (Foa et al. 2009). Beginning with the Vietnam War, and more recently with the wars in Iraq and Afghanistan (Department of Defense [DOD], 2007, p. ES-1), PTSD has been the most commonly diagnosed mental health disorder for veterans returning from combat. Epidemiological studies of Operation Enduring Freedom (OEF)/OIF veterans treated in the Department of Veterans Affairs (VA) health care system have found that 14 to 22 percent of returning veterans were diagnosed with PTSD (Seal et al. 2009; Tanelian and Jaycox 2008), making it the signature psychological wound of these two wars (DOD 2007). People are diagnosed with PTSD after exposure to a trauma if they experience a strong emotional response to the event that is followed by persistent difficulty in three key areas, including reexperiencing (e.g., nightmares, flashbacks), arousal (e.g., startle response, sleep disturbance), and avoidance (e.g., withdrawal from people, places, and other reminders of the trauma). These disruptions often lead to an impaired ability to function in social, educational, and work environments, making PTSD a very debilitating condition. More recently, research has found that PTSD and related disorders, such as depression, can develop in military personnel not only as a result of combat exposure but also as a result of childhood traumas, military sexual trauma (MST), mortuary affairs duty, and training accidents (Foa et. al. 2009).

## Military Trauma and Alcohol Misuse

Not only does military trauma increase the likelihood of developing stress-related mental health disorders such as PTSD or depression, but, as alluded to earlier, there is also evidence that traumatic experiences are related to problematic alcohol use among military members. One form of military traumatic stress that has been surprisingly under-researched is the psychological impact of exposure to killing within a combat setting. In a series of studies, Maguen and colleagues (2010*a*, *b*) examined the relationships among experiences with killing within combat and psychological adjustment of combat veterans, including problematic alcohol use. As predicted, engaging in killing during combat was related to PTSD symptoms but also was independently linked to problematic alcohol use as well as the overall quantity and frequency of alcohol use among these soldiers. These results suggest that killing within the context of combat may be a distinctive risk factor for heavy drinking and problematic alcohol use following combat among members of the military.

In addition to combat-related traumatic experiences elevating the risk for alcohol misuse, there is also evidence that MST is associated with alcohol misuse among military personnel. In a review of the literature on MST, Suris and Lind (2008) examined the relationship between MST experiences and mental and physical health outcomes. They concluded that MST was related to a variety of negative mental and physical health outcomes, including elevated rates of alcohol misuse among those who experienced MST compared with nontraumatized individuals. Taken together, these results suggest that various forms of military trauma, including exposures to killing in combat and MST, elevate the risk for problematic alcohol use among members of the military. These findings also suggest that alcohol misuse is likely to co-occur with other posttraumatic mental health disorders, such as PTSD and depression, among military personnel. Therefore, it is important to examine the co-occurrence of alcohol misuse within the context of these posttraumatic mental health disorders and to develop models that might explain these comorbidities.

## Is Alcohol Used to Self-Medicate Symptoms of Military Posttraumatic Psychiatric Disorders?

The self-medication hypothesis has been proposed to explain the relationship between military traumatic stress and alcohol use disorders. According to this model, the relationship between traumatic events and the heightened risk for an alcohol use disorders is mediated by the occurrence of PTSD or other posttraumatic psychiatric disorders (Jacobsen et al. 2001; Khantzian 1999). Specifically, traumatic events are proposed to lead to psychiatric disorders such as PTSD or depression, and individuals manifesting these conditions may turn to alcohol use as a means of “self-medicating” their symptoms. From a learning-theory paradigm, alcohol use is hypothesized to be negatively reinforcing in that it provides immediate and short-term relief from posttraumatic psychiatric symptoms. For example, military veterans with PTSD reported using alcohol to specifically cope with re-experiencing and hyperarousal symptoms (Bremner et al. 1996), and given the powerful, short-term negative reinforcement effects of alcohol, the theory postulates that people may begin to use alcohol frequently and excessively, resulting in the development of an alcohol use disorder.

Although the self-medication hypothesis proposes that the initial development of an alcohol use disorder is reactionary to PTSD or other post-traumatic psychiatric disorders, an important corollary is that alcohol abuse impedes recovery and even worsens symptoms of posttraumatic mental health disorders. Within a cognitive–behavioral paradigm that attempts to understand the necessary conditions to recover from PTSD, it is hypothesized that the individual must be able to eliminate avoidance of stressful situations—i.e., they must put themselves into contact with people, places, or things that are objectively safe but that continue to cause distress, such as being in crowds, thinking about the trauma, or experiencing emotions related to the trauma (Foa and Kozak 1986). Alcohol misuse can interfere with this necessary precondition for recovery by leading individuals to continue to engage in unhelpful avoidance behaviors. In fact, within the self-medication framework, alcohol use can in itself be conceptualized as an avoidance behavior (e.g., using alcohol to avoid thinking about the traumas). In addition, alcohol withdrawal symptoms can mirror or exacerbate the symptoms of PTSD (Jacobson et al. 2001). For example, people experiencing post–acute withdrawal may have increased irritability, sleep problems, difficulty concentrating, and anxious and depressed mood, all of which overlap with symptoms of PTSD or depression. Thus, alcohol misuse feeds back into the posttraumatic mental health symptoms, in a bidirectional manner (see the figure).

Not only do alcohol use disorders complicate recovery from posttraumatic mental health disorders, such as PTSD, but these stress-related conditions have been found to impede recovery from alcoholism. Ouimette and colleagues (1999) found that substance-dependent veterans with PTSD had poorer substance abuse treatment outcomes after 2 years compared with those without PTSD. Consistent with these results, Brown and colleagues (1999) found that substance-dependent individuals with co-occurring PTSD relapsed more quickly than those without PTSD. Taken together, these results suggest that the co-occurrence of an alcohol use disorder with PTSD provides a substantial barrier to recovery from both of these disorders.

Although large-scale research from civilian populations have found support for the self-medication hypothesis (e.g., Breslau et al. 1991), there has been less research on this theory in post–Vietnam War era samples. In a study of OEF/OIF veterans, Jakupcak and colleagues (2010) found that although combat exposure per se did not increase the risk for alcohol misuse, screening positive for PTSD or depression doubled this risk. The authors concluded that the findings may be consistent with the hypothesis that these veterans were misusing alcohol as a means of coping with symptoms of PTSD and depression. In addition, the authors found that alcohol misuse was particularly associated with emotional numbing symptoms of PTSD, suggesting that veterans may have been drinking alcohol in an effort to improve their mood or to increase emotional connectivity with others. However, because these data were collected cross sectionally, it was not possible to clearly examine the causal and temporal relationship between the development of the psychiatric symptomatology and the onset of alcohol use disorders, raising questions regarding the directionality of these relationships.

Evidence shows that PTSD is not the only stress-related condition that might mediate the relationship between stress and alcohol misuse in military personnel. In a stratified, large-scale sample of military reservists, Gradus and colleagues (2008) examined whether symptoms of depression explained the relationship between military sexual harassment experiences and alcohol misuse, and they found that more severe sexual harassment was related to greater depression symptoms among female reservists. In addition, experiencing greater amounts of sexual harassment was related to higher alcohol misuse. However, when depression symptoms were entered into the equation, the relationship between women’s experience of sexual harassment and alcohol misuse was no longer significant. These data suggest that female military reservists may be prone to abuse alcohol as a way of coping with depression symptoms that are secondary to experiencing military sexual harassment.

## Does Heritability Play a Role in Military Members’ Alcohol Misuse and Posttraumatic Psychiatric Disorders?

Research on veterans suggests that common genetic underpinnings may partially explain the relationship between combat exposure, posttraumatic psychiatric disorders, and alcohol misuse. Much of this evidence comes from studies that are derived from the Vietnam Era Twin Registry (McLeod et al. 2001; Scherrer et al. 2008; Xian et al. 2000). This registry involves a large-scale sample of monozygotic and dizygotic twin pairs who served in the military during the Vietnam era. By examining the relationships between degree of combat exposure, posttraumatic psychiatric disorders, and alcohol misuse among twin pairs that share identical (i.e., monozygotic) or nonidentical (i.e., dizygotic) genetics, researchers derived estimates as to the relative degree of genetic and environmental contributions in explaining experiences in these domains.

Several conclusions were reached by studies of the Vietnam Era Twin Registry data. PTSD and alcohol use problems were both found to be influenced by genetics, although environmental factors explained about one-half of the variance in alcohol misuse and over one-half of the variance in PTSD symptoms (McLeod et al. 2001; Xian et al. 2000). These findings suggest that although genetic factors are notable in explaining these disorders, environmental factors are equal to, if not more substantive, than genetics. Of interest, Xian and colleagues (2000) found that shared family environment did not add to the model in predicting these disorders. This suggests that environmental factors other than the family environment may be responsible for much of the variation in PTSD and alcohol misuse. In addition, these studies concluded that a common genetic element partially accounts for the co-occurrence of combat exposure, posttraumatic psychiatric disorders, and alcohol misuse. In other words, genetic factors may predispose individuals to end up in combat situations and to develop symptoms of PTSD, depression, and alcohol use disorders. Building on this finding, Scherrer and colleagues (2008) found that the genetic and environmental contributions to PTSD, in particular, explained the link between combat and alcohol misuse as well as combat and depression. This suggests that a combination of genetic and environmental vulnerability for the development of PTSD may entirely explain linkages between combat exposure and later alcohol misuse and development of depression. Such a conclusion is important because it suggests that improving understanding of the etiology of and treatment for PTSD may be a key to addressing alcohol misuse and depression following combat exposure.

## Is Alcohol Misuse a Pre-existing Risk Factor for Traumatic Stress Recovery?

Although it is possible that military members may engage in alcohol misuse as a way of trying to cope with posttraumatic psychiatric symptoms, there also is evidence to suggest that pre-existing alcohol misuse contributes to posttraumatic psychiatric maladjustment. A longitudinal study by Dickstein and colleagues (2010) found several trajectories of recovery from PTSD symptoms among U.S. soldiers who were deployed to Kosovo on a peace-keeping mission. Although most soldiers (84 percent) exhibited a resilient recovery following their deployment (i.e., low initial PTSD symptoms that decreased over time), a minority exhibited problematic levels of PTSD during the follow-up period. After controlling for other possible risk factors, higher predeployment alcohol misuse distinguished soldiers who experienced PTSD symptoms over the postdeployment follow-up period. These results suggest that problematic drinking prior to the traumatic combat experience may be a risk factor for some soldiers to exhibit PTSD symptoms following combat exposure.

Although these findings suggest that problematic alcohol use may be a risk factor that precedes the development of PTSD, they are not necessarily inconsistent with the self-medication model. Predeployment alcohol misuse may be a behavioral signal for soldiers’ pre-existing maladaptive coping strategies. For example, soldiers who misuse alcohol prior to deployment may be especially prone to abuse alcohol following deployment as a way of trying to self-medicate PTSD re-experiencing symptoms and to avoid difficult and painful emotions. This type of avoidance-based coping strategy is considered an underlying factor in the exacerbation of PTSD symptoms (Foa and Kozak 1986). Hence, these soldiers may be especially prone to attempt to self-medicate posttraumatic psychiatric symptoms, thereby worsening the course of the posttraumatic psychiatric condition.

Findings from Dickstein and colleagues (2010) that alcohol misuse is a risk factor for PTSD can also be considered from the perspective of genetics research on combat, PTSD, and alcohol misuse. As previously described, the common genetic and environmental elements that connect alcohol misuse with combat exposure seem to be those shared through PTSD (Scherrer et al. 2008). Hence, the evidence reported by Dickstein and colleagues (2010) may be attributed to the common genetic and environmental vulnerabilities that alcohol misuse shares with PTSD. In this way, predeployment alcohol misuse may be an observed indicator of an underlying latent environmental and genetic vulnerability for the development of PTSD. Clearly, additional longitudinal research is required to tease out how environmental and genetic risk factors influence the course of developing PTSD and alcohol use disorders.

## Traumatic Brain Injury, Alcohol Misuse, and Stress-Related Disorders

The causal links between alcohol misuse and posttraumatic mental health problems are further complicated by the role of traumatic brain injury (TBI) among military members. The rates of traumatic brain injury resulting from combat have increased dramatically with veterans from OEF and OIF versus veterans from prior conflicts. This increase in rates of TBI may be at least partially explained by improvements in body armor and the medical response to combat injuries. With these modern technologies, OEF and OIF veterans are now able to survive injuries that would have resulted in death in prior combat eras. However, many of these OEF and OIF veterans who now survive combat trauma are left with the repercussions of TBI. These TBI events often result from blast exposure during combat, which also can lead to post-traumatic mental health disorders (Corrigan and Cole 2008). Some studies have found that up to 44 percent of veterans who reported loss of consciousness and 27 percent of veterans who reported altered mental status also met criteria for PTSD (Hoge et al. 2008). Given this co-occurrence, defining the etiology of these presenting complaints can be difficult. Furthermore, the relationship between alcohol misuse and TBI often is complex because heavy drinking may predate and predispose individuals to experiencing a TBI (i.e., TBI can result from accidents that occur when people are under the influence of alcohol). In addition, alcohol misuse can exacerbate the complications of TBI by worsening TBI symptom severity (e.g., persistent memory problems) and by further increasing an individual’s risk for experiencing additional alcohol-related TBI events. In summary, there are likely to be multiple interrelated factors explaining the relationship between experiencing traumatic events and alcohol misuse among members of the military.

## Implications for Assessment and Intervention

Research on the self-medication hypothesis and genetic studies suggests that alcohol misuse following military trauma is likely to be highly related to the co-occurrence of PTSD and other post-traumatic psychiatric problems. Thus, early screening and identification of those who are exhibiting posttraumatic mental health problems is an important first step in intervention. In addition, given the demonstrated vulnerability for those with posttraumatic psychiatric disorders to also exhibit alcohol misuse, screening and intervention efforts should be comprehensive in addressing this common comorbidity.

Although posttraumatic psychiatric problems may be an important mediating factor between military trauma and alcohol misuse, alcohol misuse within the military is a complex phenomenon and one that is likely to have causal factors. As alluded to above, military personnel who misuse alcohol prior to experiencing military-related trauma may be prone to abuse alcohol following trauma, even in the absence of developing posttraumatic mental health problems. Thus, efforts by the military and Veterans Affairs (VA) to screen for early signs of alcohol misuse are important to identify at-risk individuals before they are exposed to combat-related trauma. As shown by Dickstein and colleagues (2010), military members who exhibit a pretrauma history of alcohol misuse may be prone to exhibit poorer recovery from PTSD symptoms following trauma exposure. Therefore, interventions to screen for a history of alcohol misuse also may help to target individuals who are at risk for developing increasingly severe PTSD symptoms following military trauma exposure.

In response to this need, the VA Healthcare System has taken extensive measures to address the issue of co-occurring substance use disorders and PTSD. For example, funding has been provided to establish substance use disorder–PTSD specialists who augment specialized PTSD treatment programs. The role of these specialists is to facilitate the assessment and diagnosis of these disorders in returning veterans and serve as a primary provider of mental health services for veterans with these comorbid conditions. Of note, a VA consensus panel (Department of Veterans Affairs 2009) recommended that specialists in these positions provide first-line evidence-based treatments such as Seeking Safety (Najavits 2002) or motivational interviewing (Miller and Rollnick 2002). The panel also recommended that substance use disorder treatment programs should continue to use empirically supported treatments focused on treating the substance use disorder. Likewise, the panel recommended that PTSD treatment programs should continue to provide evidence-based treatments targeting PTSD. Finally, the panel concluded that the superiority of any one given treatment approach above another is not supported by the literature to date and that no “gold standard” treatment exists at this time. This serves as a reminder that ample opportunities exist within the VA and military settings to further study these existing treatments and to develop alternative approaches to treating these comorbid conditions.

## Summary

Alcohol misuse is a problem among a significant minority of the U.S. military. Military-related traumatic stress seems to elevate risk for individuals to misuse alcohol. The co-occurrence of posttraumatic psychiatric disorders seems to play a major explanatory role in the association between military stress and alcohol misuse. Screening and intervention for alcohol misuse, particularly following exposure to military-related trauma, is clearly needed, as are integrated treatments that address conjoined alcohol and PTSD problems.

## Figures and Tables

**Figure:**
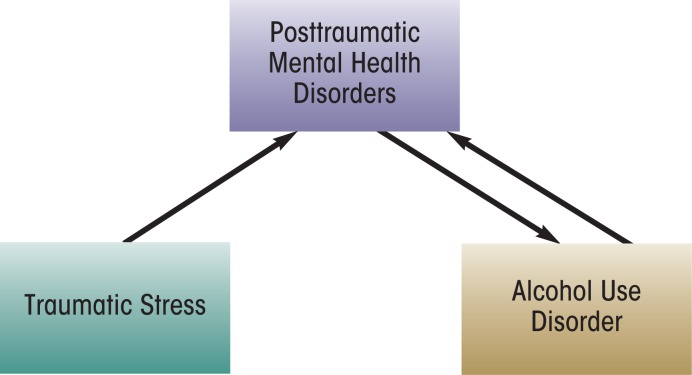
Self-medication model explaining the link between traumatic stress and alcohol use disorder.
